# Hijacking of Embryonic Programs by Neural Crest-Derived Neuroblastoma: From Physiological Migration to Metastatic Dissemination

**DOI:** 10.3389/fnmol.2019.00052

**Published:** 2019-03-01

**Authors:** Céline Delloye-Bourgeois, Valérie Castellani

**Affiliations:** University of Lyon, University of Lyon 1 Claude Bernard Lyon 1, NeuroMyoGene Institute, CNRS UMR5310, INSERM U1217, Lyon, France

**Keywords:** neural crest, neuroblastoma, migration and development, metastasis, embryogenesis

## Abstract

In the developing organism, complex molecular programs orchestrate the generation of cells in adequate numbers, drive them to migrate along the correct pathways towards appropriate territories, eliminate superfluous cells, and induce terminal differentiation of survivors into the appropriate cell-types. Despite strict controls constraining developmental processes, malignancies can emerge in still immature organisms. This is the case of neuroblastoma (NB), a highly heterogeneous disease, predominantly affecting children before the age of 5 years. Highly metastatic forms represent half of the cases and are diagnosed when disseminated foci are detectable. NB arise from a transient population of embryonic cells, the neural crest (NC), and especially NC committed to the establishment of the sympatho-adrenal tissues. The NC is generated at the dorsal edge of the neural tube (NT) of the vertebrate embryo, under the action of NC specifier gene programs. NC cells (NCCs) undergo an epithelial to mesenchymal transition, and engage on a remarkable journey in the developing embryo, contributing to a plethora of cell-types and tissues. Various NCC sub-populations and derived lineages adopt specific migratory behaviors, moving individually as well as collectively, exploiting the different embryonic substrates they encounter along their path. Here we discuss how the specific features of NCC in development are re-iterated during NB metastatic behaviors.

## Introduction

Neuroblastoma (NB) is a devastating pediatric malignancy that accounts for 15% of cancer-related deaths in childhood (Maris, 2010). NB has the particularity of being a highly heterogeneous disease, in terms of clinical outcome, ranging from spontaneous regression with little or no therapeutic intervention, to aggressive and fatal relapse despite intensive treatment. Tumor heterogeneity has been described at multiple levels. These include the anatomical localization of the tumor and its histology. The latter is mainly defined by the relative proportion of neuroblastic ganglionic cells and reactive stromal Schwann-like cells that determines the tumor’s differentiation status (Vo et al., 2014). Heterogeneity is also reflected in the genomic/molecular profile (Louis and Shohet, 2015; van Groningen et al., 2017). Two major recent studies also conjointly reported intratumor heterogeneity both at the cellular and molecular levels, opening the way to new considerations regarding the etiology and potential plasticity of NB tumors (Boeva et al., 2017; van Groningen et al., 2017).

While the scientific and medical communities have made intense efforts to better understand this complex set of malignancies and to propose new therapeutic strategies, the long-term overall survival rates remain under 50% and fall below 10% in the case of relapse (Maris, 2010). The latter mostly concerns aggressive, highly metastatic forms of the disease, which remain a major therapeutic challenge.

Anatomical localization of primary NBs, markers typically expressed by NB cells, and data obtained from mouse genetic models of the disease provided evidence that NB is a malignancy of the sympathetic nervous system, deriving from the sympatho-adrenal neural crest (NC) trunk sub-lineage (Maris et al., 2007). As such, primary tumors can arise all along the sympathetic trunk from the neck to the pelvis, within the paravertebral sympathetic ganglia and the adrenal gland. Nevertheless, recent data reporting another NC-derived source of adrenal chromaffin cells—the Schwann Cells Precursors, (SCP)—have raised the interesting possibility of an additional cellular origin for NB (Furlan et al., 2017), that we will discuss later in this review. NC-derived pathologies, namely neurocristopathies that include NB, have in common a particular etiological feature that resides in the transitory, embryonic nature of their cell of origin (Vega-Lopez et al., 2018). Neurocristopathies are depicted as developmental diseases, which is particularly relevant regarding NB malignancies that almost exclusively affect young infants as well as neonates and fetuses more rarely. The NC is a vertebrate acquisition that was described as the “fourth germ layer” on account of its potential to produce a great diversity of cell types and to form tissues and organs that are conserved among all vertebrates (Hall, 2000). This cell population has unique embryonic features, including a plethora of specialized migration strategies, that are totally or partially reiterated in NB pathology, and reflected by the biology of the disease. NC cells (NCCs) and NCC derivatives have been studied extensively with respect to their migratory capacities. Particularly striking is the robustness of the strategies they use to reach their final targets, according to their initial location, the developmental stage, and the molecular and physical structures they are confronted with during their journey. These considerations are of key importance to better understand the etiological steps and the biology of NB tumorigenesis. Indeed, sympatho-adrenal NCCs (SA-NCCs) and SCPs, the probable cells of origin of NB, show specific migratory features, some of which reflect the developmental timing of their migration. While SA-NCCs begin their journey at early developmental stages using a stereotyped short-range collective migration guided by a set of cues produced by the successive microenvironments they traverse (Gammill and Roffers-Agarwal, 2010), SCPs emerge at later stages and migrate over longer distances using preexisting physical structures—the peripheral nerves—to specifically target the innervated adrenal medulla (AM; Furlan et al., 2017). Considering the heterogeneity within (intra) and between (inter) NB tumors, and their very high and precocious potential to disseminate to specific secondary tissues, the analysis of the migratory features of their cell(s) of origin may well provide a determinant entry point to elucidate the etiology and pathogenesis of the devastating metastatic forms of the disease.

## Developmental Migration Modes of NCCs and Their Derivatives: From Physiology to Pathological Exploitation by NBs

### Migrating Like Trunk Neural Crest Cells

The NC is a transient embryonic structure that emerges during gastrulation and neurulation at the dorsal roof of the neural tube (NT), all along the rostro-caudal axis (Le Douarin and Kalcheim, 1999; Bronner and LeDouarin, 2012). The NC can be divided into four main subtypes that contribute to different anatomical territories and cell lineages: cephalic, cardiac, enteric, and trunk NC. A common trait of NCC is their extraordinary potential to migrate throughout the developing embryo, and to differentiate into a wide range of cell types suggesting an initial multipotent state (Bronner-Fraser and Fraser, 1988). Whether NCCs are predetermined before the onset of their delamination from the NT or whether they acquire specificities later on, on their road towards their final target, has long been debated and investigated in a series of studies, although the two hypotheses are not exclusive. Recent data obtained in elegant mouse models more formally demonstrated the multipotency of a large majority of trunk NCCs (Baggiolini et al., 2015). Indeed, Baggiolini et al. (2015) coupled the *confetti* mouse system with conditional transgenic lines to specifically label single clones of trunk NCCs at either premigratory or early migrating stages. *Via* this approach, trunk NCCs clones were distinguished by ten distinct colors, allowing to trace their progeny. Interestingly, both premigratory and migrating NCCs were described to maintain multipotency with only a small minority of them giving rise to single types of differentiated cells.

Trunk NCCs generate mainly neurons (sympathetic and sensory) and Schwann cells (the glia of the peripheral nervous system), that line the ventral roots of the spinal cord, chromaffin cells of the AM and melanocytes. The potential of trunk NCCs to differentiate into specific derivatives is not only dependent on their initial genetic multipotency but is also shaped by the successive microenvironments they interact with during their migration, by specific cues emanating from them, and by the physical structures they encounter upon migration (Gammill and Roffers-Agarwal, 2010).

#### Specification, Delamination/EMT of Trunk NCCs

After the neural plate borders have been induced, complex gene regulatory networks are successively involved to achieve trunk NCC specification, maintenance of their multipotent state, delamination from the dorsal NT, epithelio-mesenchymal transition (EMT), migration towards target tissues and finally differentiation into specific derivatives (Sauka-Spengler and Bronner-Fraser, 2008; Bronner and LeDouarin, 2012). In the trunk NC, a critical set of transcription factors known to be sufficient to induce NC specification and to initiate EMT, have been reported and consists of Sox9/10, FoxD3 and Snail2 (Sauka-Spengler and Bronner-Fraser, 2008). During EMT, trunk NCCs switch from having tight adhesions to neighboring neuroepithelial cells to a mesenchymal, migratory phenotype that allows them to digest the extracellular matrix and initiate migration. As highlighted in recent reviews, NCCs probably modify and degrade the surrounding extracellular matrix by upregulating the expression of several matrix metalloproteases at early steps during their delamination (Alfandari et al., 2001; Monsonego-Ornan et al., 2012; Gouignard et al., 2018). These steps are coordinated by a concomitant activation of BMP signaling and upregulation of Wnt pathway. These act in concert to drive the sequence of events characteristic of EMT. One of the key steps requires the cytoskeletal transition from type I “strong” cadherins—i.e., N-cadherin and E-cadherin—to type II “weak” cadherins—i.e., cadherins 7 and 11—(Cheung et al., 2005; Taneyhill et al., 2007). Through complex and interconnected signaling networks, these major rearrangements also affect other types of cell adhesion molecules such as occludins, connexins and integrins (Ikenouchi et al., 2003; Shin et al., 2006). NCCs also benefit from permissive substrates, principally comprised of laminin and fibronectin, to initiate and complete their migration.

#### Trunk NCC Migration and Specification of Sympatho-Adrenal NCCs

**Figure 1:**
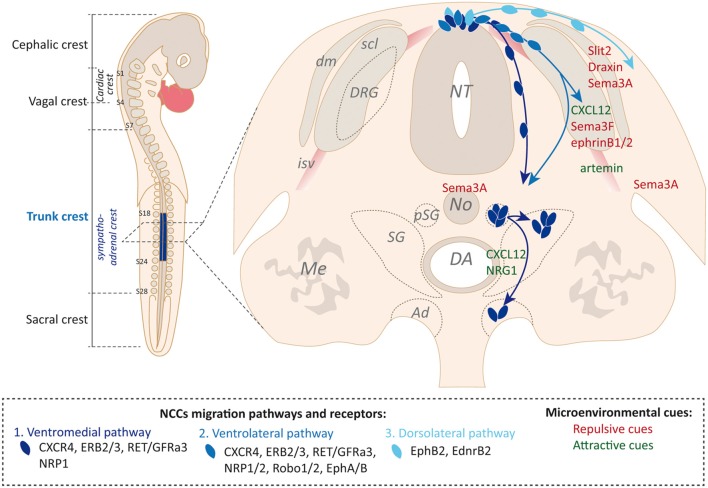
Microenvironment-driven guidance of trunk neural crest cells (NCCs) along defined migration pathways. *Left*: schematic view of neural crest (NC) subtypes along the rostro-caudal embryonic axis. Relevant somites to delimit NC populations are numbered on the scheme. *Right*: transverse section schematic view of trunk NCCs successive waves of migration, figured with blue arrows. Receptors involved in NCCs guidance interaction with environmental cues are listed for each pathway. Examples of environmental cues produced by key structural choice points are mentioned (in red, repulsive cues; in green, attractive cues). From the dermamyotome: Slit2, Draxin and Sema3A; from the posterior sclerotome: CXCL12, Sema3F and EphrinB1/2; from the Notochord and forelimb: Sema3A; from the intersomitic vessels: artemin; from the para-aortic mesenchyme: CXCL12 and neuregulin1 (NRG1). AM, adrenal medulla; DA, dorsal aorta; dm, dermamyotome; DRG, dorsal root ganglia; isv, intersomitic vessel; Me, mesonephros; NT, neural tube; Me, mesonephros; No, notochord; pSG, primary sympathetic ganglia; scl, sclerotome; SG, sympathetic ganglion.

Three major migratory routes have been described for trunk NCCs, reflecting successive waves of migration following delamination (Figure 1). Trunk NCCs first follow a ventromedial pathway in which they migrate around the developing somite, along blood vessels of the intersomitic space and between the NT and the somite. Upon somite maturation into dermomyotome and sclerotome subdivisions, trunk NCCs undertake a ventrolateral pathway in which they invade the somite in an area strictly restricted to the rostral half of the sclerotome. Finally, a last wave of trunk NCCs follows a dorsolateral pathway between the ectoderm and the dermomyotome (Gammill and Roffers-Agarwal, 2010; Krispin et al., 2010; McLennan et al., 2015). The fate of trunk NCCs is directly connected to the migratory pathway they follow. While the ventromedial pathway mainly drives NCCs towards sympatho-adrenal fates (neurons and glia of the sympathetic ganglia and chromaffin cells of the AM), the ventrolateral path will only modestly contribute to sympathetic fates, and will preferentially contribute to the dorsal root ganglia (DRG) neurons and the Schwann cells of the ventral roots (Gammill et al., 2006; Vega-Lopez et al., 2018). The dorsolateral pathway drives NCCs dedicated to the generation of pigment cells of the epidermis, although some melanocytes were also demonstrated to derive from Schwann cell precursors that have previously migrated ventrally along the nerves (Adameyko et al., 2009; Nitzan et al., 2013). The switch from the ventral to the dorsolateral migration pathway is thought to be dependent on NCC autonomous mechanisms rather than on significant changes in the NC microenvironment (Vega-Lopez et al., 2018). NCCs that have followed a ventral path and performed a first stop to aggregate into a primary sympathetic ganglion (SG), near the dorsal aorta (DA), are considered to be fate-restricted SA-NCCs progenitors (Howard, 2005; Huber, 2006). Under the influence of DA-derived signaling molecules described below, SA-NCCs then differentiate and concomitantly separate into sympathetic and adrenal medullary lineages that are destined for different territories (Reissmann et al., 1996; Saito et al., 2012). While sympathetic cells remain close to the DA, adrenal cells move further ventrally to reach the presumptive territory of the AM.

**Figure 2:**
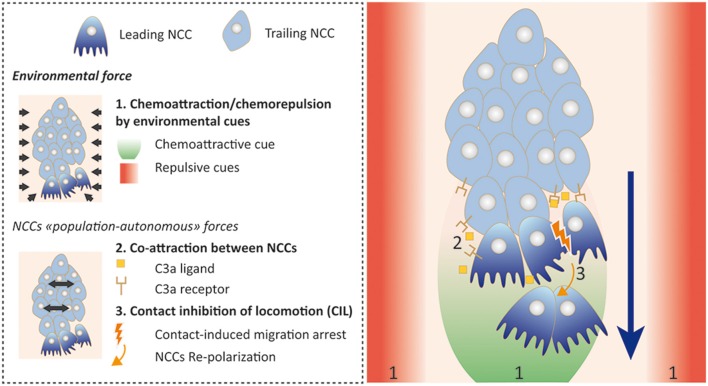
Trunk NCCs collective migration strategy. Collective migration of trunk NCCs figured as the result of combined environmental influences (environmental force, 1) and NCC population intrinsic influences (NCCs “population-autonomous” forces). The latter encompass a co-attraction mechanism (2) led by C3a ligand/receptor co-expression within the NCC stream and contact inhibition of locomotion (CIL; 3) ensuring NCCs migration stop and repolarization upon cell-cell collision. These mechanisms act in concert to drive a supracellular organization and migration of the NCCs stream.

The specification of the ventral migration pathway relies on two main mechanisms that contribute together to allow an efficient and directional migration: (1) guidance information is provided by the successively crossed microenvironments; and (2) autonomous control occurs by the NCC population itself (Figure 2).

During their long-range migration, NCCs are confronted with a series of territories that act as key choice points by presenting guidance cues to precisely orient directional migration. Trunk NCCs that underwent EMT and migrated ventrally express the CXCR4 receptor allowing them to be attracted towards the sclerotomal source of the CXCL12 guidance cue, its corresponding ligand (Belmadani et al., 2005; Gordon et al., 2011). Simultaneously, NCCs are repelled from the dorsolateral pathway by repulsive signals emanating from the dermomyotome. Here Slit2 acts as a classical repulsive guidance cue, by pushing NCCs that express Robo receptors away (Jia et al., 2005). Another well-known axon guidance cue, Draxin, produced at the dorsal lip of the dermomyotome and by the dorsal NT, may also act as an inhibitory cue but* via* a distinct mechanism. Draxin was proposed to inhibit the migration of trunk NCCs towards the dorsal path by blocking the cytoskeletal rearrangements underlying their polarization (Su et al., 2009; Zhang et al., 2017). The segmental migration of trunk NCCs within restricted somitic and peri-somitic areas is also tightly dependent on repulsive cues that mark “no-go” territories. Semaphorin 3A and 3F produced respectively by the dermomyotome and the posterior sclerotome repel trunk NCCs from these two somitic areas by acting on NRP1 and NRP2 receptors expressed at the surface of NCCs (Gammill et al., 2006; Schwarz et al., 2009a,b). This limits their migration path to the rostral part of the sclerotome. This migratory restriction is also dependent on Ephrin-B ligands produced by the caudal sclerotome which prevent NCCs that express EphA/B receptors from invading the whole somite (Krull et al., 1997; Santiago and Erickson, 2002). While moving more ventrally, SA-NCCs encounter successive major choice points that together will dictate the migratory behavior of NCCs and orient their specification. Indeed, somitic repulsive cues act in coordination with attractive cues emanating from the para-aortic mesenchyme. Again, CXCR4-expressing NCCs are attracted by the CXCL12 cue produced ventrally by cells surrounding the DA (Gordon et al., 2011). This ventral aortic attraction is further reinforced by Neuregulins that guide NCCs that express Erb2/3 receptors towards the para-aortic mesenchyme (Britsch et al., 1998; Fantauzzo and Soriano, 2015). During this migration process, Artemin, a member of the GDNF family of ligands, central to the development of the sympathetic nervous system, and produced by smooth muscle cells of blood vessels, was proposed to attract migrating sympathetic precursors, that express Ret/GFRa3 receptor complex, towards their final target (Honma et al., 2002). Finally, Semaphorin 3A sources that now surround the NCCs stream—the notochord, the forelimb and the dermomyotome—act on NRP1-expressing NCCs to arrest their migration and to induce their coalescence into primary sympathetic ganglia (pSG), near the DA (Kawasaki et al., 2002; Figure 1). The final segregation of sympathetic and adrenal medullary lineages is instructed by BMP factors produced by the DA that specifically pave a chemical pathway for endocrine precursors so that they advance ventrally and join the developing adrenal gland (Saito et al., 2012). Sympathetic precursors remain proximal to the DA and coalesce into a mature SG.

Extensive work has elucidated the collective behavior of cranial NCCs and their intrinsic mechanisms that control the global directionality of the stream. While the molecular effectors specifically controlling trunk NCC collectivity are mostly unknown, several experimental observations suggest that most of the mechanisms described for cranial NCCs are also driving trunk NCCs to their ventral target sites (Dyson et al., 2018; Gouignard et al., 2018). Live video microscopy experiments, mainly performed on chick and *Xenopus Laevis* embryos, revealed sheets of migrating trunk NCCs, tightly connected to each others and showing the features of supra-cellular dynamic organization typical of collective migration processes (Krull et al., 1997; Kasemeier-Kulesa et al., 2005). Cranial NCC migration is the result of simultaneous: (1) chemotactic attraction/repulsion involving the traversed microenvironments; (2) co-attraction between NCCs; (3) contact inhibition of locomotion (CIL); and (4) repolarization of leading and trailing cells within the migratory stream (Theveneau and Mayor, 2012). Co-attraction of migrating trunk NCCs was recently demonstrated to be dependent on Complement 3a (C3a) ligand co-expressed with the C3a receptor by NCCs in the stream, as previously described for cranial lineages (Broders-Bondon et al., 2016; Figure 2). Nevertheless, recent data show that trunk and cranial NCC migration differ with respect to their behavior within the collectivity. Cranial NCCs are not assigned to leading or trailing positions within the migrating stream. They can intermingle and adopt either role transiently. On the contrary, in chains of migrating trunk NCCs, leader and follower cells are predetermined before the onset of migration and remain physically and functionally assigned to their initial position (Richardson et al., 2016). These observations could underlie differences between trunk and cranial NCCs regarding their multipotentiality and the associated migratory strategy each NCC subtype uses to drive the stream.

#### Neuroblastoma Cells as Tumoral SA-NCCs?

The localization of NB primary tumors all along the sympathetic chain and within the AM, the expression of SA lineage markers and the deregulation of some well-known SA-NCC molecular actors in NB cells established the idea that NB cells of origin are SA progenitors. Furthermore, a number of factors involved in the different steps of SA-NCCs development, ranging from NC induction to SA specification, have been implicated in NB tumorigenesis and extensively reviewed elsewhere (Tomolonis et al., 2018; Tsubota and Kadomatsu, 2018). We will focus here only on the effectors of the migration machinery involved in SA-NCC behavior to highlight those used and/or hijacked by NB cells in the tumorigenic process.

Many parallels regarding the unique potential of NCCs to migrate and metastatic cancer cells and cancer stem cells, have been discussed in the literature (reviewed recently in Gallik et al., 2017). As such, EMT and migration of NCCs during development and in cancer invasion show striking similarities, both in terms of strategies and the molecular players. Regarding NB, that has a direct ontogeny relationship with NCCs, data converge onto the idea that these embryonic cancer cells exploit or hijack NCC characteristics and especially their molecular interplay with the embryonic microenvironment throughout the tumorigenic process.

A key example resides in the EMT process that is central to the initiation of NCC migration and that is determinant in the NB invasive phenotype. FoxD3, Slug and Sox9/10 constitute the minimal inducers of EMT and have all been shown to be dysregulated in aggressive NBs. Slug/Snail2 was found hyperactivated in a range of NB cell lines *via* activation by the transcription factor c-myb (Tanno et al., 2010). Overexpression of Snail2 maintains NB in a mesenchymal, invasive state, favoring NB dissemination. Comparative analyses of trunk NCCs and their NB pathological counterparts allowed LMO4 to be identified as an essential co-factor of Snail2, mediating E-Cadherin repression and boosting tumor invasiveness (Ferronha et al., 2013). FoxD3 was depicted as a tumor suppressor with decreased expression in NB primary tumors and cell lines. Experimental knockdown of FOXD3 was shown to have a direct positive impact on NB cell migration and invasion, notably *via* its effect on matrix metalloproteinase 9 (MMP9) expression, but also on NB cell growth and angiogenesis suggesting that it has a general effect on the tumorigenic process (Li D. et al., 2013). A role of long non-coding RNAs (lncRNA) was also evidenced by a recent study demonstrating that FOXD3 anti-sense RNA (FOXD3-AS1) is decreased in NB tumors. *In vitro* assays and xenografts in *nude* mice revealed that FOXD3-AS1 harbors tumor-suppressive properties by inhibiting the oncogenic roles of PARP1 or CTCF (Zhao et al., 2018). MMPs are themselves found upregulated in advanced NB stages, favoring degradation of extracellular matrix components and enhancing tumor cell metastatic migration (Ara et al., 1998; Ribatti et al., 2001).

The above EMT-related tumorigenic mechanisms relate to NB metastatic dissemination, after the primary tumor has already formed in sympatho-adrenal derivatives. Our team recently showed, in addition, that stage 4 NB cell lines and patient samples share with NCCs the ability to migrate in a collective, stereotyped manner within the embryonic microenvironment, to reach the primary tumor site (Delloye-Bourgeois et al., 2017). Human NB grafts in the dorsal hedge of the NT, in the avian embryo, at the sympatho-adrenal NC levels, revealed that NB cells migratory behavior is coordinated both in time and space with that of endogenous SA-NCCs. Once confronted by this specific embryonic microenvironment, NB cells undergo a typical “in chain” or “en masse” collective migration. They avoid endogenous SA-NCC no-go territories and coalesce at specific SA-NCC target sites, mainly in the sympathetic ganglia and the AM. Thus, NB cells retain the ability to read and respond to the cues produced by the microenvironments they encounter. We further observed that the experimental knockdown of the guidance cue Semaphorin 3C in NB cells perturbed NB collectiveness within the migratory stream. This interfered with their ability to attain sympatho-adrenal target tissues.

Comparative transcriptomic data obtained from this NB avian model revealed that in the primary tumor, profound modifications of gene expression occur as a result of contact with the sympathetic microenvironment to prepare NB cells for secondary dissemination. Among the major molecular and cellular functions related to these changes, genes involved in cell migration, adhesion, invasiveness and axon guidance were found to predominate. In particular, we identified a pathological microenvironment-induced drop in the Semaphorin 3C/Neuropilin/PlexinA4 signaling pathway within NB cells of the primary tumor, that leads to a loss of cell-cell cohesion. This switch of adhesion participates in the onset of the metastatic process, and illustrates a key role of embryonic microenvironment exploitation by NB cells in the pathological process. The Semaphorin 3C receptor, Neuropilin 1 was itself found downregulated in NB with poor prognosis (Delloye-Bourgeois et al., 2017; Ishizuka et al., 2018). Neuropilin 1 knockdown increased NB migratory and invasive abilities, and was associated with an increase in Integrin β1 expression (Ishizuka et al., 2018).

Several other key guidance signaling pathways underlying SA-NCCs stereotypical migration have been associated with NB invasive properties. The CXCR4/CXCR7/CXCL12 axis is one of the first examples, and has been involved in the organ-specific dissemination of a range of cancer types. High levels of CXCR4 expression are detected in aggressive stages of NB and correlate with a worse prognosis (Russell et al., 2004; Zhang et al., 2007). Together with CXCR7, CXCR4 chemokine receptor activity, that is shared with migrating NCCs, seems to be hijacked by NB cells to selectively support their growth in the adrenal gland and NB secondary homing to the liver and bone marrow (BM; Mühlethaler-Mottet et al., 2015). Assays performed on tail-vain injections of human NB cell lines in immune-deprived mice suggested a combined mode of action of these two receptors in NB dissemination towards organs and tissues that release CXCL12 ligand, including the liver and BM. As CXCR4, ERBB2/3 receptors are implicated in the attraction of NCCs towards ventral paths and linked to NB tumorigenesis. The impact of their expression levels and biological function with respect to tumor growth and metastasis remains complex to interpret and translate to the clinic (Richards et al., 2010; Izycka-Swieszewska et al., 2011). The guidance cue Slit2 has itself been involved in NB cells motility (Huang et al., 2011). Upon pathological overexpression of the neuronal differentiation factor NeuroD1 in NB sympathetic tumors, Slit2 expression is silenced, boosting NB cell migratory potential. These observations correlate with clinical data showing that high expression of NeuroD1 in NB is associated with a poor prognosis. No direct role for EPH receptors and Ephrin-B ligands has yet been identified in NB tumorigenesis. However, several studies show that their co-expression in NB correlates with low grade disease, suggesting an implication in the control of NB tumorigenesis (Tang et al., 2000a,b).

### Migrating Like Neural Crest-Derived Schwann Cells Precursors

SCPs are embryonic glial progenitors that directly originate from NCCs and are associated with nerve fibers. They are considered to be a cell population intermediate between NCCs and immature Schwann cells. The transition from NCC to SCP identity is not fully clear, from either a phenotypical point of view (Dupin and Sommer, 2012) or when considering their respective genetic programs (Jessen et al., 2015). NCCs are considered to be SCPs as soon as they are observed expressing Sox10 and found associated with nerves. SCP functions during nerve development in the growing embryo remain unclear in most cases.

Very recent reviews of novel convergent data (Dupin et al., 2018; Furlan and Adameyko, 2018), have shed light on unexpected non-canonical functions of SCPs both during embryogenesis and adulthood. We will focus here on the migratory strategies employed by SCPs to efficiently and durably supply developing or injured organs with a reservoir of multipotent cells.

#### SCP Migratory Strategy and Contribution to Neural-Crest Derived Tissues

The association of SCPs with developing nerves lies at the heart of key molecular interplays that sustain SCP survival, proliferation and maintenance of their multipotency, a phenotypical trait that they share with NCCs. SCP survival is dependent on Neuregulin ligands produced by the axons on which SCPs migrate. Neuregulins act on SCPs *via* ERBB2/3 receptors expressed at their surface. Neuregulin1 (*Nrg1*) heterozygous mutant mice show myelination defects, consistent with a loss of myelinating Schwann cells (Michailov et al., 2004). The Neuregulin/ERBB pathway acts in coordination with the JAGGED1/NOTCH pathway showing similar *in trans* ligand/receptor expression profiles in SCPs and nerves, and boosting SCP proliferation. SCPs themselves were proposed, in specific contexts, to sustain the functional and parallel architecture of peripheral nerves and blood vessel networks. This was documented in the embryonic skin, where CXCL12 and VEGF produced by SCPs instruct the differentiation of vessels into arterioles and venules, resulting in parallel development of both networks (Li W. et al., 2013).

The event of SCP emergence as determined by the detection of nerve-associated cells expressing Schwann cell-specific genes, is assumed to arise slightly later during embryogenesis than the onset of NCC migration, at least in the mouse embryo. Whether this desynchronization is linked to cell autonomous and/or microenvironmental changes is not clear. However, in the light of the divergent migratory strategies adopted by these two embryonic multipotent cell populations, their coexistence can be considered to be an efficient way to supply developing target organs and to coordinate their respective contributions to the establishment of complex tissues within a fast-growing embryo (Furlan and Adameyko, 2018). Another consideration regarding the divergent roles of NCCs and SCPs is the possibility to maintain a local, and long-lasting pool of multipotent cells in close proximity to a given organ.

Nerve-free collective migration of NCCs at early developmental stages in both dorso-lateral and ventral pathways is an efficient strategy to populate presumptive organ territories such as the skin, the sympathetic ganglia and the AM (Furlan and Adameyko, 2018). Upon establishment of nervous connections and with the increased size and complexity of embryonic tissues, switch to an NCC migration mode along nerves can be seen as an adaptive strategy to supply specific innervated target tissues with precursors and to achieve coordinated building. Such a local recruitment *via* the innervation network was first demonstrated for SCP-derived melanocytes, to supply internal pigmented organs such as heart, inner ear or brain meninges with pigment cells (Adameyko et al., 2009, 2012). Interestingly, SCPs lying on presynaptic fibers were proposed to generate progenitors of the corresponding post-synaptic parasympathetic ganglia (Dyachuk et al., 2014; Espinosa-Medina et al., 2014). This typically illustrates the fundamental role of SCPs in coordinating the embryonic developmental timeline with the functions of the respective networks and organs.

**Figure 3:**
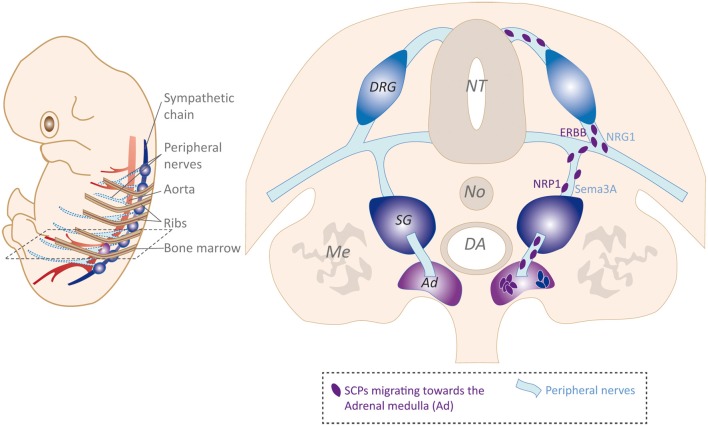
Schwann cells precursor (SCP) migration strategy, example of SCPs supplying the adrenal medulla (AM) with chromaffin cell precursors. *Left*: schematic view of embryonic closely related sympathetic chain and connected peripheral nerves, DA, and ribs. *Right*: transverse section schematic view of SCPs emerging from the NC and undergoing perineural migration to reach the AM as a final target. NRG1/ERBB ligand/receptor *in trans* interaction is presented as a hypothetic signaling mediating SCPs/nerve interaction.

A recent study brought new insights concerning the development of the sympathetic and adrenal lineages. By performing lineage tracing experiments in mouse models that allowed chromaffin cells originating from glial cells to be distinguished from those derived from “early” migrating NCCs, Furlan et al. (2017) concluded that more than 80% of adrenal chromaffin cells originate from migrating SCPs (Figure 3). Even if not exclusive regarding previous results, these data overturn the historical view of a unique common sympatho-adrenal progenitor. The authors could also document the involvement of preganglionic fibers innervating the AM in efficiently supplying SCPs, and confirmed the divergent origin of most adrenal chromaffin cells as opposed to that of sympatho-adrenal progenitors undergoing an earlier, nerve-free migration in the developing embryo. This breakthrough study was further supported by Lumb et al. (2018), who confirmed the requirement of preganglionic axons for chromaffin cell precursors to colonize the adrenal primordia. Furthermore, they identify type 3 Semaphorins and their receptors Neuropilins 1 and 2 as major actors allowing the guidance of axons to the AM and the maintenance of a close association between chromaffin cell precursors and preganglionic nerve fibers (Lumb et al., 2018; Figure 3).

Using the nerves as a niche to proliferate and a route to reach specific organs, SCPs have selected an efficient and long-lasting perineural migratory strategy. These considerations bring new insights into cancers that could derive from these particular progenitors.

#### Are NB Cells Tumoral SCPs?

As already suspected for non-cutaneous melanomas (Hussein, 2008), the recent data obtained by Furlan and collaborators, point to SCPs as potential cells of origin for “NC-derived” cancers. NB primary tumors occur in the adrenal gland in more than 50% of cases. Therefore, the SCPs that supply the developing adrenal gland with chromaffin progenitors are very likely candidates for being additional NB cells of origin. As primary NB tumors also develop in the sympathetic chain, these considerations bring the idea that sympathetic and adrenal NB could differ in their etiologic history. Consistently, adrenal and non-adrenal NB tumors show distinctive clinical, biologic and prognostic features (Vo et al., 2014). Given the extreme heterogeneity of NB tumors mentioned above, the coexistence of two (or more) distinct cells of origin of the disease could, at least in part, contribute to the diversity of clinical patterns observed. Possibly, the two cell-types provide distinct origins for either sympathetic or adrenal primary NBs. Alternatively, each precursor could also contribute to both tumor sites, and/or SA-NCCs supplying sympathetic ganglia and AM in specific proportions could be the common cellular source of all NBs.

Our team recently documented early metastatic routes undertaken by NB cells that detach from the primary tumor. Interestingly, using the avian NB xenograft model, we could observe that, once primary tumors have developed, NB cells undergo a secondary dissemination and follow a migratory strategy that totally differs from the one of early migrating SA-NCCs (Delloye-Bourgeois et al., 2017). NB cells colonize the adjacent aorta but also the peripheral nerves proximal to the adrenal gland and the sympathetic ganglia. While these two migration paths have to be characterized in more detail, the perineural migration strategy selected by NB cells to disseminate towards secondary sites could reflect their phenotypic similarity with SCPs. An interesting starting point would be to follow this study by correlating the anatomical localization of NB primary tumors formed in the avian model to the path they use for undergoing metastatic dissemination.

### Contributing to the Bone Marrow Niche Like NCCs and NCCs Derivatives

The migratory potential of NCCs and their immediate derivatives such as SCPs has been reported to contribute to a previously unsuspected derivative, the fetal BM, that fills the cavity of the developing bones. The BM is the primary site of hematopoiesis, all blood cells being derived from hematopoietic stem cells (HSCs) that mostly reside in the BM. Recent research has provided an integrated and complex picture of the BM microenvironment, also called “stem cell niche,” and of its multiple and dynamic contributions to the regulation of HSC differentiation and functions (Agarwala and Tamplin, 2018; Wei and Frenette, 2018). Interestingly in the context of the present review, NCCs were reported to contribute, directly or indirectly, to two subsets of cells lying in the BM niche: HSCs themselves at early stages of specification (Bertrand et al., 2010; Boisset et al., 2010; Kissa and Herbomel, 2010; Damm and Clements, 2017) and mesenchymal stem cells (MSCs) that will ensure stromal support functions in the niche (Isern et al., 2014).

#### NCCs and NCCs Derivatives Contribution to the Bone Marrow Stem Cell Niche

**Figure 4:**
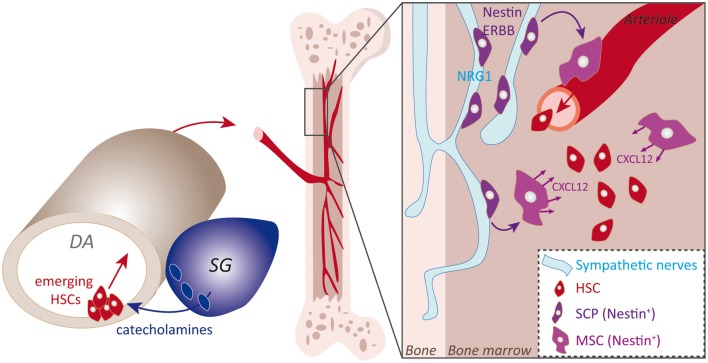
Sympatho-adrenal NCCs (SA-NCCs) and SCPs contributions to the bone marrow (BM) niche. Schematic representation of NC main contributions to the BM niche. SA-NCCs addressed next to the ventral portion of the aorta induce the hematogenous ventral aortic endothelium to generate HSCs, an induction that might involve catecholamines produced by SA-NCCs. Emerging HSCs then colonize the BM niche *via* the blood circulation. The second described mechanism resides in NCCs-derived SCPs that colonize the BM niche *via* perineural migration on sympathetic nerves that innervate the niche. SCPs then give rise to Nestin^+^-MSCs that mainly sustain HSCs by secreting CXCL12 cue.

Trunk NCCs pursuing a ventral pathway stop their migration in close contact with the DA endothelium. A series of studies have concluded that some of the HSCs residing in the BM, the lineage founders of the adult hematopoietic system, originate from the hemogenic ventral endothelium of the DA (Bertrand et al., 2010; Boisset et al., 2010; Kissa and Herbomel, 2010). Several lines of evidence suggest that trunk NCCs conveyed adjacent to the aortic wall contribute to the hemogenic activity and to the early specification of HSCs (Figure 4). For instance, recent data showed that by specifically blocking Pgdf receptor signaling in trunk NCCs, which is assumed to be involved in their targeting next to the aortic endothelium, NCCs migration to the aorta is completely disrupted. This defect was found to be associated with a profound alteration of HSCs specification (Damm and Clements, 2017). However, the specific signal provided by trunk NCCs to initiate HSC specification has not been formally identified, even if the literature converges on the idea that catecholamines, neurotransmitters produced by trunk NCC derivatives, positively regulate the emergence of aortic HSCs (Fitch et al., 2012; Kwan et al., 2016; Damm and Clements, 2017; Figure 4). Interestingly, Nagoshi et al. (2008) had previously performed *in vivo* experiments to trace the migration of multipotent NCCs and observed the presence of labeled NCCs next to the aorta (Aorta-Gonad-Mesonephros, AGM), along blood vessels within the BM and also in the fetal liver. These observations strongly suggested that NCCs could themselves migrate to the BM by reaching the aorta and the blood vessels connected to the niche.

The second identified contribution of NCCs to the BM niche is to provide a direct supply of NCC-derived stromal MSCs during fetal stages. Several subsets of MSCs reside in the BM and play key specific roles in establishing or sustaining the different specialized BM lineages. Lineage tracing experiments pointed to a subset of MSCs, which express Nestin, and have a common origin with sympathetic peripheral neurons and Schwann cells that are also part of the BM niche (Isern et al., 2014). Interestingly, as with SCPs, Nestin-positive MSCs undergo a perineural migration along developing peripheral nerves that allows their specific targeting to the BM niche (Figure 4). Again, the neuregulin receptor ERBB3 is essential for MSCs to migrate along nerves. Indeed, ERBB3 constitutive knockout severely impairs this process while sparing peripheral nerve development (Riethmacher et al., 1997). This results in a decrease in the number of MSCs in the fetal BM niche (Isern et al., 2014). NC-derived MSCs then secrete the CXCL12 chemokine which stimulates the colonization of the nascent niche by HSC (Ding et al., 2012). Indeed, the seeding of HSCs to the BM is drastically reduced upon CXCL12 experimental blockade or upon perinatal deletion of Nestin^+^ cells. Thus, the spectrum of SCP-derived fates is not limited to the nervous system but also includes mesenchymal fates. Interestingly, this diversification of fates is coupled to dedicated migratory paths. Overall this set of data illustrates the wide range of migratory strategies of NCCs and their close interplay with key biological systems of the body.

#### Do NB Cells Hijack NCC Modes of Contribution to the BM Niche During Tumorigenesis?

Widespread rapid colonization of the BM is one of the typical traits of aggressive NB and is central is the mechanism of relapse. The physical routes undertaken by metastatic NB cells to reach the BM have never been formally documented *in vivo*, which also coincides with the clinical fact that metastases to the BM are already present at diagnosis, even in very young infants. The newly reported contributions of NCCs and/or SCPs to the BM niche, at early stages of its formation, provide new hypotheses regarding the metastatic routes underlying NB colonization of the BM. Whether NB cells exploit the periaortic niche and the factors produced by endogenous trunk NCCs to select this major embryonic vessel as a pathway has not been clarified yet. Our team has observed a very rapid invasion of the DA by NB cells, soon after the formation of sympatho-adrenal primary tumors within the NB avian model (Delloye-Bourgeois et al., 2017). It is thus tempting to speculate that the particular microenvironment that trunk NCCs contribute to is not only perceived by stem cells of the hemogenic aortic endothelium but also by closely affixed NB cells. Similarly, the peripheral nerve pathway followed by NCC-derived MSCs to populate the BM fetal niche also appears to be a highway from which they may profit. This strategy would enable NB cells to quickly and efficiently attain this territory at early developmental stages. If validated, these possibilities would highlight new aspects of the etiology of NB metastasis, that occur during the early embryonic emergence of the disease.

## Metastatic Neuroblastoma: Switch From an Embryonic-Like Condition to a Malignant Metastatic Disease

These diverse scenarios regarding NB cell(s) of origin are not mutually exclusive and share major common points that reside in the extreme multipotency of these embryonic lineages and in the adaptive migratory mechanisms that they adopt over their developmental history. These particular properties echo the *in vivo* observations we made using our experiments in the NB avian xenograft model. When grafted in the embryonic organism, metastatic NB cells, both from cell lines and patient samples, reiterate a physiological NCC-like initial migration to target the sympathico-adrenal territories (Delloye-Bourgeois et al., 2017). This suggests that NB cells maintain the gene program that supports this physiological migration in a plastic state and can reactivate it when needed. Then, the collective NCC-like migratory behavior is switched off in the primary tumor to allow consecutive migration strategies that mimic SCP and NCC behaviors and ensure metastatic dissemination *via* the aorta and the peripheral nerves.

The cellular and molecular mechanisms underlying these changes are not known yet, but recent data exploring the basis of NB heterogeneity shed light on potential regulations that could sustain such adaptive modifications of NB cells in the successive environments they meet. Indeed, the existence of an intra-tumor heterogeneity in NB has been clearly documented and fits with long-term clinical data reporting the existence of different types of NB cell lines distinguished by their morphology and molecular markers. NB cell lines were historically classified as “N type” for neuroblastic; “S type,” for non-neuronal Schwann cell-like; or “I type” for morphologically Intermediate (Ciccarone et al., 1989). van Groningen et al. (2017) were able to generate these different types of cell lines from the same patient. The same team made the demonstration that two types of cells coexist within NB tumors: mesenchymal cells with an NCC-like identity (MES-type) and committed cells with a sympathetic noradrenergic identity (ADRN-type). The latter observations were confirmed and extended by Boeva et al. (2017). Both studies concluded that rather than based on different genetic abnormalities, these two cell types are distinguished by distinct super-enhancer landscapes and associated transcription factors. These studies established key epigenetic mechanisms underlying intratumor heterogeneity and bring new insights into the potential plasticity of NB cells within the primary tumor. The panel of epigenetic regulations contributing to the NB disease remains poorly characterized, although several additional illustrations have been provided in recent years (Chen et al., 2018; Li et al., 2018). It will be interesting to determine whether epigenetic mechanisms control the adaptive migration/dissemination behavior of NB cells.

## Stage 4S Neuroblastoma: A Developmental Disorder With Physiological NCC-Derived Migration Modes

### Stage 4S NB: Unique Clinical and Biological Features of Unknown Origin

Stage 4S (S, for special) NB is a highly particular and enigmatic form of disseminated NB that occurs only in neonates, usually before 1 year of age. Tumor foci can be numerous and are typically detected in the liver, under the skin, and/or in the BM but without skeletal involvement. Masses considered to be primary tumors are located in sympatho-adrenal derivatives (van Noesel et al., 1997). Despite this wide dissemination profile already present at diagnosis, stage 4S NB usually have a very good prognosis that contrasts with stage 4 aggressive cases. This is mainly attributable to the rapid spontaneous regression of tumor foci with very little or no therapeutic intervention (Brodeur, 2018). These major differences between stage 4 and stage 4S NBs have raised many questions regarding their respective etiology, genomic, epigenetic and transcriptomic specificities. Studies performed on rare cohorts of stage 4S patients did not reveal discriminating genomic features such as genomic abnormalities. For example whole chromosomes gain or loss are similar between Stage 4 and Stage 4S (Bénard et al., 2008). Stage 4S specificities were finally identified from their molecular and epigenetic portraits (Bénard et al., 2008; Decock et al., 2016) opening new areas of research regarding the mechanisms contributing to their unique biology and propensity to regress spontaneously.

### What Can We Learn From Developmental Modes of Migration to Understand the 4S NB Pathology?

As described above, trunk NCCs and SCPs adopt specific trajectories to populate anatomical sites that coincide exactly with tumor foci locations in stage 4S NB: sympatho-adrenal tissues, liver, skin, and BM to a lesser extent. These observations led to the hypothesis that rather than a real metastatic disease with primary tumor followed by metastatic dissemination, stage 4S NB could indeed be a multifocal pathology arising from early NCC progenitors (van Noesel, 2012). Undefined events occurring in multipotent NCCs would induce developmental aberrations leading to a transitory, mild tumorigenic phenotype. Abnormally proliferating transformed NCCs would join their target site, and excessive cellularity would be eliminated by apoptosis or would persist and be subsequently directed towards differentiation.

Although still completely speculative, this idea fits well with: (1) the very early occurrence of stage 4S NB in infants; (2) the anatomical location of tumor foci; and (3) the clinical observation that sympatho-adrenal tumors are similar in size to non-sympathetic tumor foci.

Difficulties to conduct functional studies on low grade NBs mainly reside in the absence of *in vitro* and *in vivo* experimental models. Indeed, obtaining cell lines from these clinical samples has failed for many years. Attempts to create genetically-engineered or xenograft-based animal models have also been unsuccessful or insufficiently documented. These failures can be explained by the vulnerability of low stages NB, once removed from their original microenvironment, and by the fact that causal tumorigenic mechanisms including their precise cellular origin remain uncharacterized.

The avian NB model that we recently set up could bring new insights both for NB stage 4S and localized cases. We have modeled the formation of localized NBs by performing xenografts of stage 1–2 NB patient tumor biopsies. As stage 4 NB, NB cells from localized tumors follow an NCC-like ventral pathway leading to the formation of dense, proliferating sympatho-adrenal tumors (Delloye-Bourgeois et al., 2017). Interestingly, the model also reproduces their “localized” status, since these primary tumors never switched towards a secondary dissemination. This model offers the opportunity to study the etiological steps of all NB stages. It would be an interesting challenge to engraft stage 4S NB patient biopsies in the avian model and assess their migratory behavior in the embryonic microenvironment. This should at least allow the determination of whether they disseminate towards the typical sites in which tumors form in neonates directly from the dorsal roof of the NT, where NCC progenitors originate, or *via* the typical metastatic scheme, undergoing secondary dissemination to these sites.

## Concluding Remarks

**Figure 5:**
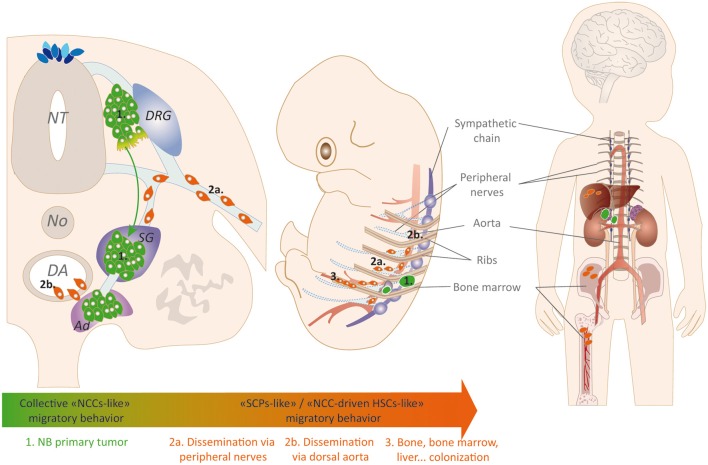
NCCs/SCPs migratory strategies potentially used or hijacked by neuroblastoma (NB) cells. NB cells are able to engage a physiological trunk NCCs-like collective migratory behavior that address them to sympatho-adrenal derivatives where they coalesce and form primary tumors (1). Within the primary tumor, NB cells preparing for secondary dissemination switch towards SCPs-like (2a) and/or NCCs-driven HSCs-like (2b) migratory behaviors that would allow them colonizing secondary foci such as the BM niche by hijacking peripheral nerves and aorta major migratory highways. Such mechanisms could explain the rapid, extensive and concomitant emergence of metastatic foci in young children affected by the disease.

Recent insights into NC-derived functions and developmental contributions have opened new perspectives regarding NB metastatic processes. Considering NB in the light of its embryonic origin(s) makes a crucial link between the extreme heterogeneity of the pathology and the complex cellular and molecular mechanisms that drive NC-related NB cell(s) of origin towards their target tissue (Figure 5). Our increasing knowledge of the vast panel of migratory strategies used by NCC and their derivatives during development suggests key similarities with NB cells. Investigating NB behaviors in light of their links with their physiological counterparts and of the cellular and molecular programs orchestrating the embryonic development complements studies of their malignant features. Such an approach could indeed help precisely determining the mechanisms hijacked by tumor cells to ensure the very early and efficient dissemination of the disease in young infants.

## Author Contributions

CD-B and VC wrote the manuscript and designed the figures.

## Conflict of Interest Statement

The authors declare that the research was conducted in the absence of any commercial or financial relationships that could be construed as a potential conflict of interest.
